# The Sulfation Code of Tauopathies: Heparan Sulfate Proteoglycans in the Prion Like Spread of Tau Pathology

**DOI:** 10.3389/fmolb.2021.671458

**Published:** 2021-05-20

**Authors:** Dylan Mah, Jing Zhao, Xinyue Liu, Fuming Zhang, Jian Liu, Lianchun Wang, Robert Linhardt, Chunyu Wang

**Affiliations:** ^1^Department of Biological Sciences, Department of Chemistry and Chemical Biology, Center for Biotechnology and Interdisciplinary Studies, Rensselaer Polytechnic Institute, Troy, NY, United States; ^2^College of Food Science and Nutritional Engineering, China Agricultural University, Beijing, China; ^3^Harvard Medical School, Harvard University, Boston, MA, United States; ^4^Eshelman School of Pharmacy, University of North Carolina at Chapel Hill, Chapel Hill, NC, United States; ^5^Morsani College of Medicine, University of South Florida, Tampa, FL, United States

**Keywords:** Alzheimer’s disease, heparan sulfate, glycobiology, 2-O and 6-O sulfated heparins, tauopathies, 3-O sulfation, prions and prion disease, neurodegenerative diseases

## Abstract

Tauopathies are a heterogenous family of progressive neurodegenerative diseases defined by the appearance of proteinaceous lesions within the brain composed of abnormally folded species of Microtubule Associated Protein Tau (tau). Alzheimer’s Disease (AD), the most common tauopathy, is the leading cause of cognitive decline among the elderly and is responsible for more than half of all cases of senile dementia worldwide. The characteristic pathology of many tauopathies—AD included—presents as Neurofibrillary Tangles (NFTs), insoluble inclusions found within the neurons of the central nervous system composed primarily of tau protein arranged into Paired Helical Fibrils (PHFs). The spatial extent of this pathology evolves in a remarkably consistent pattern over the course of disease progression. Among the leading hypotheses which seek to explain the stereotypical progression of tauopathies is the *prion model*, which proposes that the spread of tau pathology is mediated by the transmission of self-propagating tau conformers between cells in a fashion analogous to the mechanism of communicable prion diseases. Protein-glycan interactions between tau and Heparan Sulfate Proteoglycans (HSPGs) have been implicated as a key facilitator in each stage of the prion-like propagation of tau pathology, from the initial secretion of intracellular tau protein into the extracellular matrix, to the uptake of pathogenic tau seeds by cells, and the self-assembly of tau into higher order aggregates. In this review we outline the biochemical basis of the tau-HS interaction and discuss our current understanding of the mechanisms by which these interactions contribute to the propagation of tau pathology in tauopathies, with a particular focus on AD.

## Introduction

### Tau Protein

**FIGURE 1:**
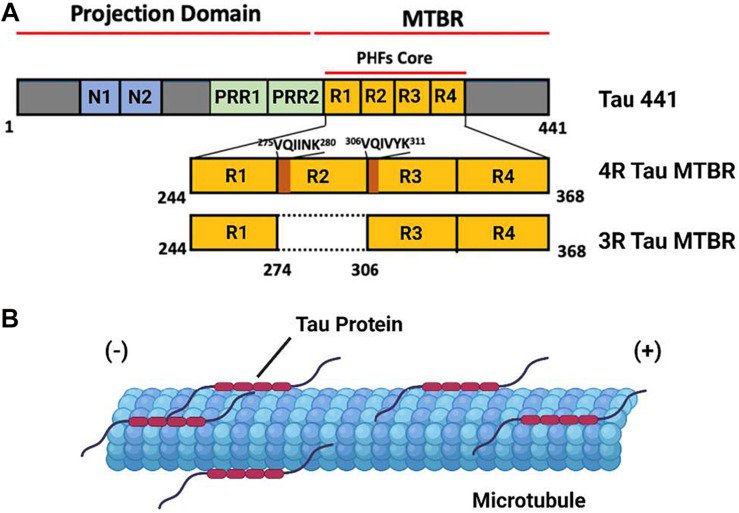
The domain Structure and Function of Tau Protein. **(A)** A domain map of the tau 441 isoform and a comparison of the microtubule binding domain (MTBR) of 4R and 3R tau isoforms. The VQIINK and VQIVYK sites implicated in tau aggregation are highlighted in dark orange. **(B)** Under non-pathological conditions, tau protein associates with axonal microtubules and stabilizes the microtubule against depolymerization.

Tau protein is the primary constituent of the proteinaceous lesions that characterize tauopathies, a group of debilitating neurodegenerative diseases exemplified by Alzheimer’s Disease (AD), the most abundant tauopathy and the leading cause of dementia in the elderly worldwide. Tau is a Microtubule (MT) binding protein encoded by the *MAPT* gene on chromosome 17 (Binder et al., 2005). Six distinct isoforms of this protein are produced in the Central Nervous System (CNS) of adult humans through alternative splicing of *MAPT*, the largest of which consists of a 441 residue polypeptide. Tau 441 consist of an N-terminus projection domain with two inserts (N1 and N2), a Proline Rich Region (PRR) subdivided into PRR1 and PRR2 which contains major tau phosphorylation sites, and an MT Binding Repeat domain (MTBR) composed of four imperfect repeat motifs (R1–R4) that participates in both MT binding and tau aggregation (Figure 1A). The CNS tau isoforms are distinguished by the presence of both (2N), one (1N), or neither (0N) of the N terminus inserts and the presence (4R) or absence (3R) of the second of the four MTBR repeats found in Tau 441, giving rise to a total of six isoforms: 2N4R, 1N4R, 0N4R, 2N3R, 1N3R, and 0N3R. According to this convention, Tau 441 is alternatively referred to as 2N4R tau (Buée et al., 2000; Goedert et al., 1989).

Tau protein is an Intrinsically Disordered Protein (IDP) which lacks a defined secondary or tertiary structure in solution. The binding of tau to a microtubule is mediated by a conformational shift toward a more ordered structure, a feature consistent with the behavior of other IDPs. Under ordinary physiological conditions, tau protein localizes to the axonal segment of the neuronal cytoskeleton, where it interacts with the tubulin heterodimer to stabilize MTs and promote tubulin polymerization (Figure 1B). The function of tau is regulated by a range of post translational modifications, including phosphorylation, acetylation, and methylation, many of which directly modulate its interaction with microtubules (Cleveland et al., 1977; Mukrasch et al., 2007; Barbier et al., 2019).

### Tau Pathology in AD and Other Tauopathies

**FIGURE 2:**
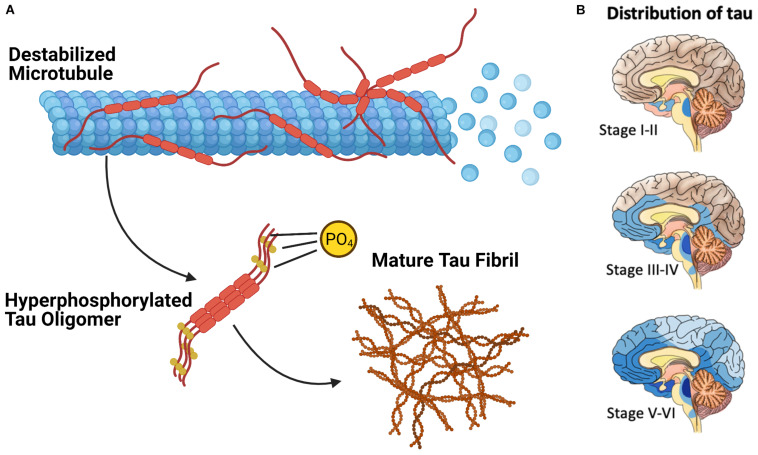
The Pathological Deposition of Tau Protein in Alzheimer’s Disease. **(A)** The Phosphorylation (denoted with “P”) of tau protein is associated with the destabilization of the tau microtubule complex and the assembly of tau protein into higher order aggregates. **(B)** Mature tau fibrils (in blue, darker color denotes greater fibril density) spreads throughout the brain of AD patients in discrete stages which resembles the progression of communicable prion diseases.

Extensive phosphorylation of tau is associated with the self-assembly of tau monomers into higher order aggregates (Figure 2A; Grundke-Iqbal et al., 1986; Goedert et al., 1996; Amniai et al., 2009; Alonso et al., 2010). Under the pathological conditions associated with tauopathies, these aggregates take the form of misfolded oligomers and filaments that spread throughout the brain in an orderly and stereotypical fashion (Mudher et al., 2017; Goedert et al., 2017b). The characteristic pattern of tau pathology in AD is described by Braak Staging (Braak and Braak, 1991), which begins with the appearance of initial tau lesions in the transentorhinal cortex during stage I. During the subsequent stages of disease progression, the density of tau lesions increases and NFTs spread to the entorhinal cortex in stage II, then to limbic regions of the brain in stage III, before finally reaching the neocortex in stage IV and beyond (Figure 2B). The progression of tau pathology is accompanied by increases in both phosphorylated and total tau in cerebrospinal fluid (CSF), and correlates remarkably well to the severity of neurodegenerative symptoms (Arriagada et al., 1992; Buerger et al., 2006).

AD is a secondary tauopathy, which can be distinguished from primary tauopathies by the presence of additional species of proteopathic hallmarks beyond the characteristic tau-based inclusions. In the case of AD, this consists of extracellular plaques composed of Amyloid β (Aβ), a ∼40 residue peptide derived from the integral membrane protein APP, while Dementia with Lewy Bodies (DLB) is characterized by extracellular inclusions composed of α-synuclein in addition to tau tangles. However, this demarcation is not absolute. For example, despite being categorized as primary tauopathies, the various forms of Frontotemporal Dementia (FTD) often present with inclusions composed of TDP-43. Additionally, a number of studies have suggested a high prevalence of tau pathology in cases of so-called mixed dementia in which a patient simultaneously exhibits hallmarks of multiple neurodegenerative pathologies. Another point of distinction that can be drawn between tauopathies are the populations of cells which exhibit tau lesions. Whereas tau pathology in AD primarily affects neurons, other tauopathies are characterized by the presence of additional tau inclusions within glial cells (Table 1). Interestingly, tauopathies also differ in terms of the predominant isoform composing their tau inclusions (Table 1), with AD exhibiting a roughly 2:1 ratio of 4R to 3R tau (Goedert et al., 2012; Wagshal et al., 2015; Irwin, 2016; Custodio et al., 2017; Josephs, 2017; Ferrer, 2018).

**TABLE 1:** Features of notable tauopathies.

**Clinical diagnosis**	**Type and localization of tau pathology**	**Additional proteopathic hallmark(s)**	**Predominant tau isoform(s)**
Alzheimer’s disease (AD)	NFT pathology in neurons	Plaques composed of aggregated Aβ peptide	3R + 4R tau
Pick’s disease	Pick’s bodies within neurons, extensive glial tau inclusions	N/A	3R tau
Corticobasal Degeneration	Coiled inclusions within neurons + glial inclusions	N/A	4R tau
Argyophillic grain disease	Spindle shaped neuronal inclusions	N/A	4R tau
Globular glial Tauopathy	Globular inclusions within astrocytes + oligodendrocytes	N/A	4R tau
Chronic traumatic Encephalopathy	NFT pathology in neurons + astrocytic tau inclusions	High frequency of TDP-43 pathology	3R + 4R tau
Primary age related Tauopathy	NFT pathology in neurons	Absence of Aβ plaques	3R + 4R tau
Dementia with Lewy Bodies (DLB)	NFT pathology in neurons	Lewy bodies of aggregated α-synuclein + tau	3R + 4R tau
Progressive Supranuclear Palsy (PSP)	NFT Pathology + coiled oligodendrocyte inclusions	N/A	4R tau
Frontotemporal Dementia (FTD)	NFT pathology + mixed glial inclusions	∼50% exhibit TDP-43 pathology	Varies

### Heparan Sulfate and HSPGs in the Prion Like Spread of Tau Pathology

A substantial body of evidence has established that the spread of tau pathology in the brain occurs through a prion-like mechanism in which seeds of pathological tau are transmitted between cells and nucleate the misfolding of physiological tau in a process known as template misfolding (Brettschneider et al., 2015). The mechanisms of transcellular tau propagation are understood best in the context of AD, where tau pathology is transmitted through the synaptic junction via the secretion of tau seeds by a presynaptic neuron and their subsequent reuptake by post-synaptic neurons (Goedert et al., 2017b; Mudher et al., 2017; Wang et al., 2017). This pathway has been shown to be mediated by Heparan Sulfate (HS) (Holmes et al., 2013; Christianson and Belting, 2014), a ubiquitous polysaccharide found across virtually all metazoans, from rudimentary invertebrates to humans.

**FIGURE 3:**
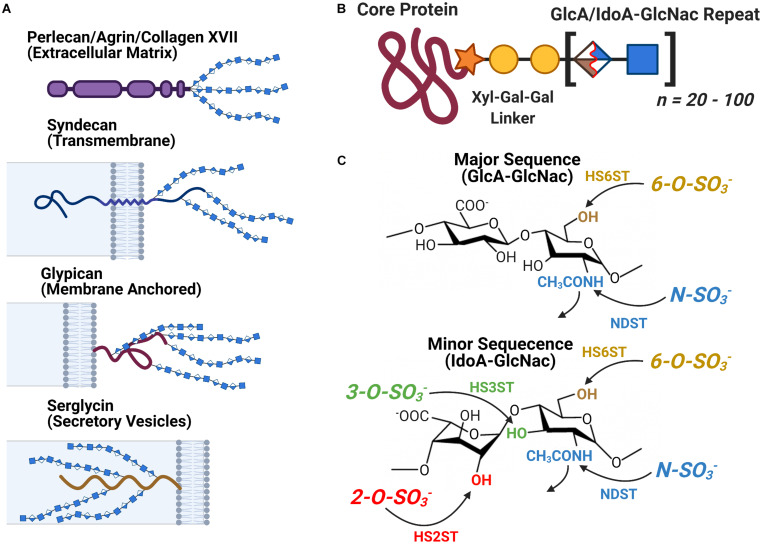
The Structure and Localization of Heparan Sulfate and its Proteoglycans. **(A)** The different families of HSPG core proteins exhibit a range of distinct localizations. **(B)** An HSPG consists of a core protein covalently attached to several HS glycan chains, each consisting of between 20 to 100 HS disaccharide units. **(C)** The chemical structure of the major and minor HS disaccharide repeats. The glycan’s variable sulfation sites are noted along with the name of the corresponding family of sulfotransferases.

Heparan sulfate is a linear Glycosaminoglycan (GAG) often encountered in the form of Heparan Sulfate Proteoglycans (HSPGs) a protein-glycan conjugate which consist of one of several families of HSPG core protein covalently attached to a series of HS chains ranging in length from approximately 20 to 120 disaccharide subunits (Figure 3). The localization of an HSPG is dictated by the identity of its core protein: perlecan, agrin, and type XVIII collagen based HSPGs are found in the extracellular matrix, syndecan and glypican based HSPGs localize to the exterior of the plasma membrane, and serglycin based HSPGs localize to the interior membrane of secretory vesicles (Figure 3A). The HS polymer is built from repeating disaccharides of either glucuronic (GlcA) or iduronic acid (IdoA) followed by *N*-acetylglucosamine (GlcNac) with the former (GlcA-GlcNac) being the more prevalent of the two. HS biosynthesis is carried out at the Golgi, during which the growing HS chain can be acted upon by several groups of enzymes to yield a variety of final structures: C5 Epimerase converts GlcA-GlcNac disaccharides to IdoA-GlcNac, while HS sulfotransferases *N*-deacetylase/*N*-sulfotransferase (NDST) and 2*-O-*, 3*- O-*, and 6*-O-*sulfotransferases (HS2ST, HS3ST, and HS6ST) carry out the sulfation of specific sites on the HS disaccharide (Figure 3C). Following synthesis, HS can be further catabolically modified by the endosulfatases Sulf1 and Sulf2, which selectively remove 6*-O-*sulfo groups from cell surface HSPGs. Heparin is a structural isoform of HS secreted by mast cells, notable for its medical use as an anti-coagulant. It is frequently used as an analog for HS to study the molecular interactions of HS *in vitro* due to its widespread availability (Capila and Linhardt, 2002; Esko and Selleck, 2002; Bishop et al., 2007; Xu and Esko, 2014; Li and Kusche-Gullberg, 2016).

Heparan sulfate proteoglycans are known to participate in endocytosis via multiple canonical pathways. In pinocytosis, extracellular molecules bind to HSPGs and are internalized directly via a clatherin and caveolin independent pathway. Depending on the size of the ingestion, this process can be subdivided into micropinocytosis, which involves the internalization of small particles, and macropinocytosis, which facilitates the uptake of larger macromolecular complexes. In receptor mediated endocytosis pathways, HSPGs often act as a cofactor which facilitate the initial rapid capture of a ligand and coordinate its subsequent binding to its receptor (Christianson and Belting, 2014). Current evidence also indicates the existence of novel, non-vesicular secretory pathways which are mediated by HSPGs; however, this process remains poorly understood at this time (Prudovsky et al., 2008; Merezhko et al., 2018; Park et al., 2020).

### Tau Determinants of Tau-HS Interaction

HS and heparin are both capable of directly binding to tau monomers, oligomers, and fibrils *in vitro*. This interaction also occurs *in vivo*, as the GAG sidechains of HSPGs are observed to colocalize with tau-based lesions in the brains of patients suffering from both AD and other tauopathies (Snow et al., 1990; Su et al., 1992). HS/tau binding is driven predominately via electrostatic interactions that occur between positively charged residues in tau and the negatively charged sulfo groups present on the HS GAG. Further work has identified the ^275^VQIINK^280^ hexapeptide present at the start of the R2 repeat of the MTBR domain as a major site of contact between tau and heparin. The interactions occurring at this site and the corresponding ^306^VQIVYK^311^ hexapeptide at the start of the R3 repeat have been shown to promote the formation of a local extended β-conformation that serves as a nucleation site for tau protein aggregation (Smet et al., 2004; Mukrasch et al., 2005; Sibille et al., 2006).

### Heparan Sulfate in Tau Protein Secretion

An emerging line of evidence has implicated Tau/HS interactions as a driver of both the secretion of tau into the extracellular space and its subsequent internalization by other cells of the CNS. Although tau protein is predominately an intracellular protein, there exists a small pool of extracellular tau even under ordinary physiological conditions (Goedert et al., 2017a; Yamada, 2017; Pérez et al., 2019). A small portion of this tau protein is present in extracellular vesicles, however, the vast majority of tau has been shown to be membrane free (Wegmann et al., 2016; Yan et al., 2016). Recently Merezhko et al. reported that the secretion of membrane free tau occurs in an ATP independent fashion, suggesting the use of a novel vesicle independent secretory pathway. The group also found that this process was abolished by treatment with heparinase—an enzyme which degrades HS—or inhibition of the HS biosynthetic pathway, indicating the participation of HS in facilitating tau protein’s entry into the extracellular space (Katsinelos et al., 2018).

## Distinct HS Sulfation Patterns Govern Tau-HS Interaction and Uptake

**FIGURE 4:**
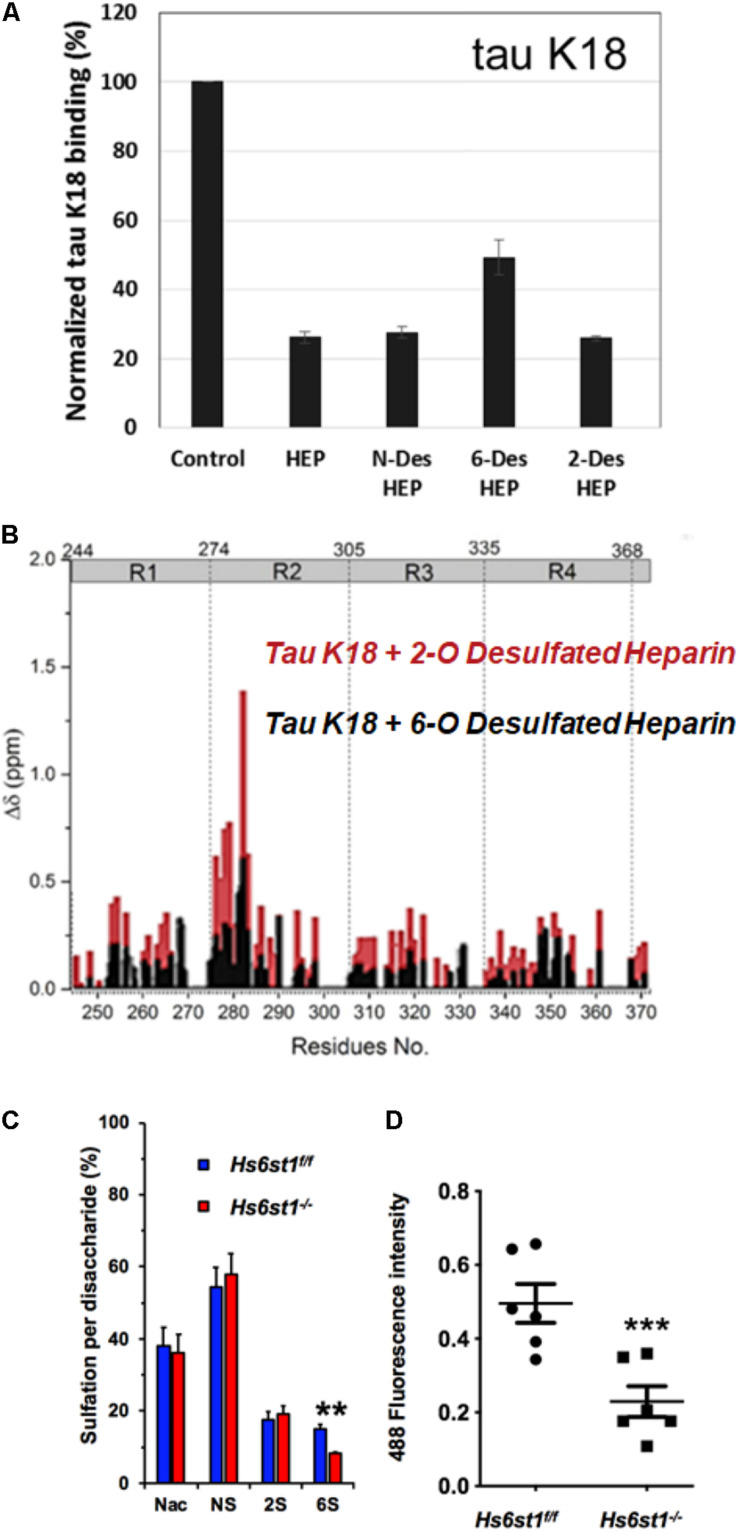
Evidence For the Role of 6-*O* Sulfation in HS Tau Binding. **(A)** Chemical removal of 6-O sulfo groups inhibits the ability of heparin to compete for K18 tau (a truncated construct consisting of the 4R MTBR of tau) binding in an SPR competition experiment compared to other sulfo- groups. In this SPR competition experiment, heparin or its analog in solution inhibits the binding between tau and heparin immobilized on the SPR chip. Heparin (HEP) and N-desulfated and 2-O-desulfated HEP can inhibit tau-heparin binding at the same level, while 6-O-desulfated HEP does not inhibit binding as efficiently. **(B)** NMR studies indicate that K18 tau exhibits significantly greater Chemical Shift Perturbation (CSP) when exposed to 2-*O* desulfated heparin compared to 6-*O* desulfated heparin, indicating the importance of 6-O sulfation in tau/HS interactions. The knockout of a 6-O HS sulfotransferase (*Hs6st1*) in lung endothelial cells significantly reduces **(C)** the amount of 6-*O* sulfated HS and **(D)** the uptake of fluorescently labeled Tau. Adapted from Zhao et al. (2017, 2020).

**FIGURE 5:**
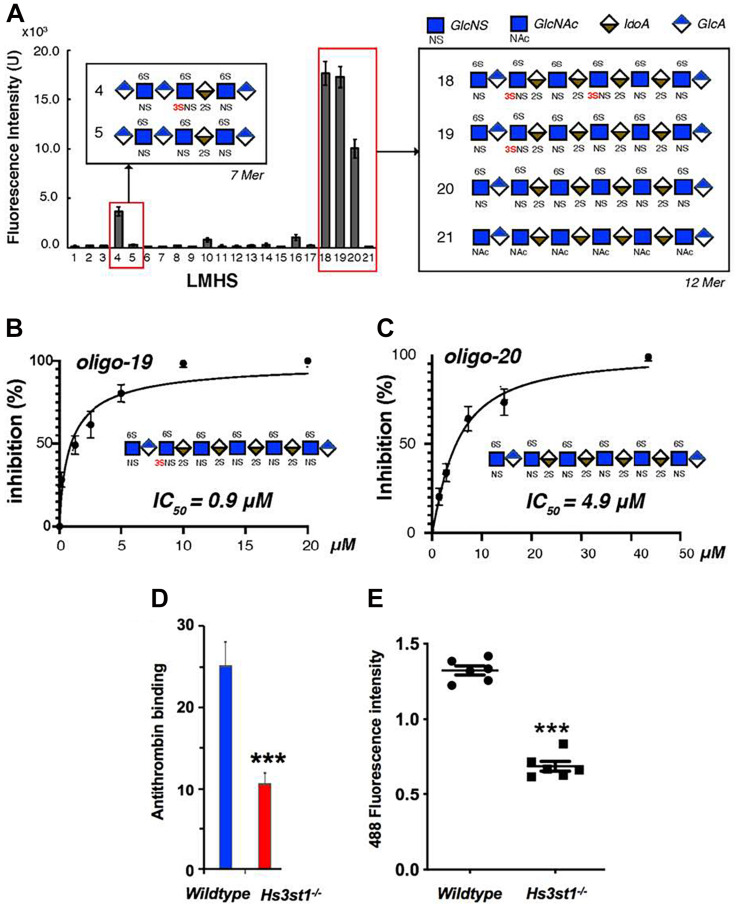
Evidence for the Role of 3-*O* Sulfation in HS Tau Binding. **(A)** A low molecular weight heparan sulfate (LMHS) binding array shows 3-O sulfated glycans exhibit enhanced tau binding compared to otherwise identical structures. Oligosaccharides **(B)** 19 and **(C)** 20 from the LHMS differ only by a single 3-O sulfo group, yet exhibit a more than 5 fold difference their in inhibition constants as measured by competition SPR. **(D)** Knockdown of *Hs3st1* in mice lung endothelial cells significantly reduces the binding of antithrombin—a canonical 3-O-S binding protein—to HS **(E)** and the uptake of fluorescently labeled tau compared to wild type cells. Adapted from Zhao et al. (2020).

**FIGURE 6:**
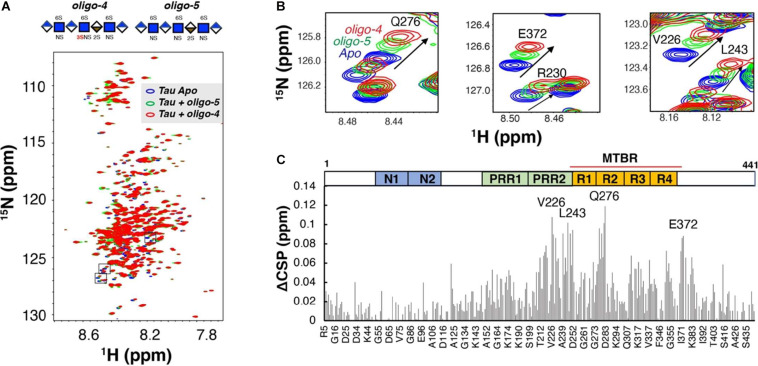
Binding of Tau to Defined 3-O Sulfated Glycans as Measured by NMR. **(A)** An overlay of two ^1^H-^15^N heteronuclear single quantum coherence spectra of full-length tau before (blue) and after 1:0.6 molar ratio addition of a 3-O sulfated HS oligosaccharide (oligo-4, green) and an otherwise identical oligosaccharide lacking a 3-O sulfo group (oligo-5, red). **(B)** Zoomed-in NMR spectra of residues with the largest Chemical Shift Perturbation (CSP). **(C)** The Comparison CSP differences of the two oligosaccharides (ACSP) reveals specific interaction between 3-0-S and tau in the residues of the PRR2 and MTBR domains of tau 441. A Domain map of tau is shown above the figure. PRR, proline-rich region; MTBR, microtubule binding repeat. Adapted from Zhao et al. (2017, 2020).

It was initially assumed that the interactions between tau and GAGs—and between GAGs and proteins in general—were non-specific and driven exclusively by the GAG’s overall degree of sulfation, making the glycan’s individual sulfation sites essentially interchangeable for one another (Capila and Linhardt, 2002). However, contrary to such expectations, our group demonstrated in 2017 substantial differences in the strength of tau/heparin interaction following the chemical removal of different sulfation moieties, indicating that 6-*O* desulfation significantly reduced tau-heparin binding, while the impact of 2-*O* desulfation was limited (Figure 4A; Zhao et al., 2017). In the same study, using a truncated tau construct consisting of the 4R MTBR domain known as tau K18, we characterized the binding of tau to heparin, 2-O desulfated heparin, and 6-*O* desulfated heparin using solution NMR. Our results were indicative of significant 6-*O*-S mediated contacts in the R2 subdomain of tau (Figures 4B–D; Zhao et al., 2017). Stopschinski et al. (2018) characterized the HS binding of various proteins involved in neurodegeneration and showed that the preference for specific sulfation moieties is particular to tau protein, with both Aβ and α-synuclein exhibiting a significantly higher degree of promiscuity for sulfation moieties than tau protein. Rauch et al. (2018) also reported a crucial role for 6-*O* sulfation of HSPGs and HS 6-O sulfotransferases in this pathway. Work by Sepulveda-Diaz et al. (2015) indicated that HS3ST2, one of the 3-*O*-S HS Sulfotransferases, acts as a crucial mediator of tau phosphorylation, suggesting a potential link between tau pathology and 3-*O* sulfated HS. Our group later identified that tau protein exhibits a direct and specific interaction with the 3-*O*-S moiety, and further implicated 3-*O*-S as a driver of cellular uptake of tau protein (Figure 5; Zhao et al., 2020), making tau one of only a handful of proteins known to interact with the rare 3-*O*-S moiety (Thacker et al., 2014). In the same paper, we employed solution NMR to compare the chemical shift perturbation induced by tau’s interaction with two chemically defined HS heptamers which differed from one another only by a 3-*O*-S moiety. Our results suggest that the major sites of contact between tau and the 3-*O*-S moiety are localized to the PRR2 domain and the R2 repeat of the MTBR (Zhao et al., 2020; Figure 6).

**FIGURE 7:**
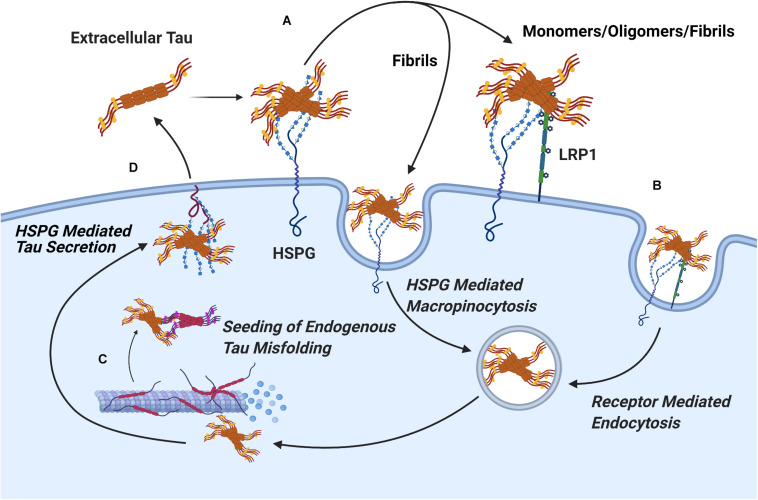
Pathways of HSPG Mediated Secretion and Uptake of Tau Protein. **(A)** The binding of tau aggregates to cell surface Heparan Sulfate is a crucial, initial stage in the cellular uptake of tau in both receptor mediated and receptor independent uptake of tau. **(B)** Mature tau fibrils readily undergo receptor independent uptake via HSPG mediated pinocytosis, while uptake of tau monomers and oligomers proceeds via receptor mediated endocytosis through the HSPG dependent receptor LRP1. **(C)** Upon uptake, extracellular tau oligomers nucleate the phosphorylation and aggregation of endogenous tau protein through template misfolding. **(D)** Interactions between tau oligomers and HSPGs mediate vesicle free secretion of misfolded tau through a poorly understood mechanism.

The current evidence concerning the mechanism of HSPGs in tau protein uptake suggests a role for two distinct pathways. A significant portion of large tau aggregates such as mature fibrils undergo direct internalization by HSPGs via macropinocytosis, while soluble oligomers and tau monomers are dependent on receptor mediated endocytosis to cross the cell membrane (Holmes et al., 2013). Consistent with this, in a recent report by Hudák et al. (2019) syndecan associated HSPGs were linked to the uptake of tau fibrils via a clathrin independent endocytosis mechanism mediated by lipid rafts, as would be expected for macropinocytosis of tau fibrils. Work in CNS cell lines has identified the probable receptor involved in endocytosis of soluble tau monomers and oligomer species as LRP1, a lipoprotein receptor which acts cooperatively with HSPGs (Figure 7; Rauch et al., 2020).

The identification of LRP1 as a receptor for tau protein uptake raises several exciting possibilities. LRP1 is a canonical receptor for lipoproteins, notably Apolipoprotein E (ApoE), the protein product of the *APOE* gene, a major risk factor for AD (Holtzman et al., 2012). ApoE has previously been shown to directly interact with tau protein *in vitro* (Strittmatter et al., 1994; Bachmeier et al., 2014), which suggests the possibility of a ternary interaction between tau, ApoE, and HSPGs which could greatly impact the course of tau pathology.

There exists tentative evidence for alternative pathways of tau protein uptake which do not utilize HS. Perea et al. (2019) reported that the uptake of monomeric tau by primary astrocytes was unaffected by pre-treatment of the cultures with heparin or heparinase, indicating the astrocytes took up tau via an as of yet unidentified HSPG independent pathway. In a comparative study of the uptake of brain derived tau oligomers from patients with AD, PSP, and DLB, Puangmalai et al. (2020) found that the knockout of the HSPG biosynthetic enzyme exostin-2, as well as treatment with HSPG antagonists abolished the uptake of AD and DLB derived tau oligomers as expected. However, uptake of PSP derived oligomers was merely slowed, suggesting the presence of an HSPG independent mechanisms of uptake specific to PSP derived tau oligomers.

### Role of Heparin and HS in Tau Aggregation

Heparin and other polyanions are capable of inducing the assembly of unphosphorylated tau protein into fibrils (Kampers et al., 1996; Wilson and Binder, 1997), and thus heparin has seen wide-spread use in *in vitro* studies of tau fibrilization. However, a significant body of evidence indicates this does not truly reflect *in vivo* aggregation. Cryo-EM studies have consistently shown that the morphology of heparin induced tau fibrils differ from those found in the brains of patients suffering from tauopathies (Fitzpatrick et al., 2017; Falcon et al., 2018; Zhang et al., 2019). This is further supported by the recent work of Despres et al. (2019) who found that heparin nucleated tau fibrils exhibited a different conformation and activity from fibrils which were seeded by *in vitro* phosphorylated tau and brain derived tau. Using solid state NMR Savastano et al. (2020) characterized the structure of truncated tau and polypeptide constructs from the PRR2 subdomain of the PRR, and found evidence for the incorporation of PRR2 into the rigid core of tau PHFs following heparin induced fibrilization, consistent with our own work indicating extensive contacts between tau and heparin in the PRR domain.

Work by Fichou et al. (2018) found that fibrils derived from both recombinant tau protein and mouse brains could be induced to depolymerize back into oligomers and monomers through the removal of polyanions. Using nanopore based sensors, Giamblanco et al. (2020) monitored tau protein during heparin induced fibrilization, comparing the FTD associated P301L tau isoform to wild type tau, and found that the P301L mutation promoted the assembly of tau monomers into oligomers, and the dissociation of tau fibrils into oligomers. In light of the evidence that tau oligomers exhibit higher cytotoxicity compared to fibrils or monomers (Tian et al., 2013; You et al., 2019), this suggests altered interactions between P301L tau and HS may contribute to familial FTD by destabilizing fibrils which would otherwise sequester tau protein and mitigate tau protein proteotoxicity. A study by Townsend et al. (2020) of the aggregation kinetics of heparin nucleated tau revealed that differential desulfation of heparin dramatically altered the kinetics of heparin induced aggregation of a recombinant tau fragment. 2-*O* desulfation was found to substantially increase the time required for aggregation and increase the flexibility of the resulting fibrils compared to those induced to aggregate with 6-*O* desulfated or N-acetylated heparin. This indicates that despite its relative weak contribution to HS-tau binding, 2-*O* sulfation of HS plays an important role in tau aggregation.

### Recent Evidence for the Importance of Tau-HS Interactions in Tauopathies

The role of HSPGs in the prion-like spread of tau pathology has gained additional support in recent years from the analysis of tissues from the brains of patients with tauopathies and other forms of *in vivo* evidence. Two meta analyses of genome wide association studies for AD risk factors have implicated enhanced expression of the 3-O HS sulfotransferase gene *Hs3st1* as an AD risk factor, supporting existing observations on the role of 3-*O* sulfated HS in tau protein uptake and phosphorylation (Witoelar et al., 2018; Schwartzentruber et al., 2021). Consistent with *in vitro* work suggesting enhanced HS-tau binding promotes enhanced spread of tau pathology, Huynh et al. (2019) have reported an increase in HS expression in the brains of patients with AD both in absolute terms and relative to chondroitin sulfate (CS), another member of the GAG family. The group also reported that AD derived HS exhibited a higher binding capacity for tau protein compared to HS derived from healthy brains, indicating AD associated changes in HS enhance the strength of its interactions with tau protein. Lorente-Gea et al. (2020) conducted a systematic analysis of major HSPG core protein expression across the regions of the brains of patients with AD. While changes in the expression of extracellular HSPG core proteins were limited in AD brains, the study revealed upregulation of cell surface syndecan and intracellular serglycin. In particular, there is extensive overexpression of syndecan 4 and serglycin, which are associated with both amyloid and tau pathology in most AD brain samples (Lorente-Gea et al., 2020), indicating a potentially undiscovered role for intracellular HSPGs in tauopathies. Some strides have been taken in recent years toward clinical translation of this line of research into potential drugs for AD and related dementias. Weisová et al. (2019) studied the mechanism of AX004, a therapeutic antibodies known to inhibit tau uptake *in vivo*, and determined the antibody’s mechanism was driven by disruption of the tau-HS interaction via binding to the MTBR. Stopschinski et al. (2020) recently reported a synthetic heparin like oligosaccharide capable of disrupting cellular propagation of tau protein at similar activity to full length porcine derived heparin. In the long term, drugs targeting the tau HS interaction could prove to be a novel therapeutic for AD.

## Summary and Outlook

**FIGURE 8:**
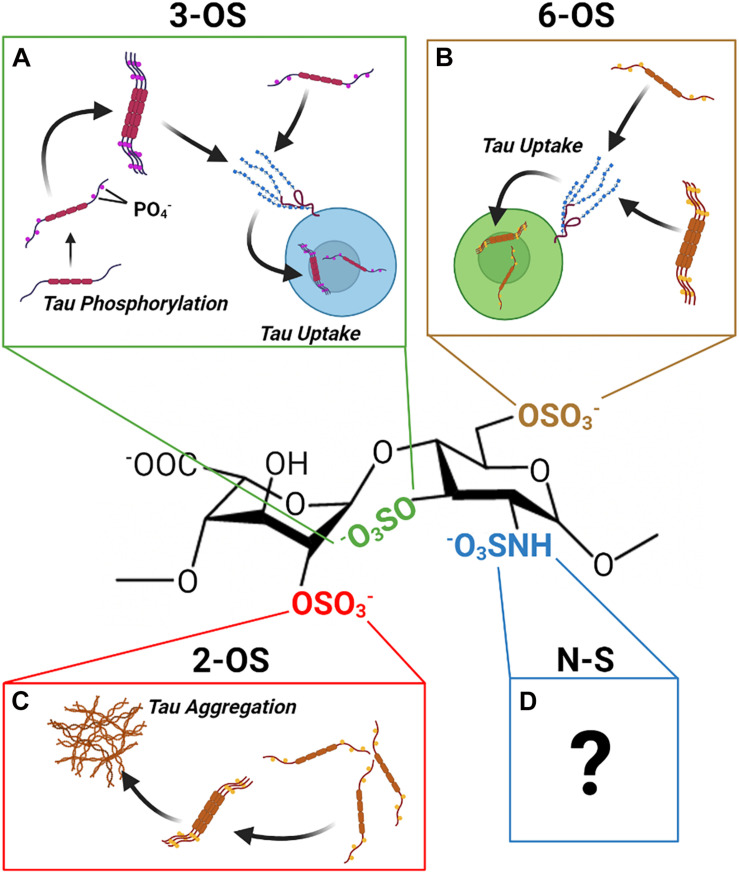
The Discrete Functions of HS Sulfation Sites. The different HS sulfation sites are associated with different interactions with tau protein. **(A)** 2-*O* HS sulfation may promote aggregation of tau. **(B)** 6-*O* HS Sulfation enhances tau protein HS binding and HSPG mediated uptake of tau. **(C)** 3-*O* HS Sulfation is associated with enhanced tau phosphorylation as well as enhanced tau binding and uptake. **(D)** Though tau exhibits an interaction with N-sulfated HS, its functional significance remains unclear.

Recent work by our group and others has revealed, contrary to prior expectations, that the sulfation sites present on the HS disaccharide exhibit specific interactions with tau protein that are not functionally interchangeable. Distinct roles in mediating HS tau interactions relevant to the aggregation, uptake, and phosphorylation of tau have been established for 2-*O*, 6-*O*, and 3-*O* sulfation, respectively (Figure 8). Multiple studies have established that direct interactions with HSPGs drive tau protein uptake in tauopathies, and emerging evidence is also suggestive of a role for HSPGs in tau protein secretion. Research into the specific HSPG core proteins involved in HSPG tau interactions is limited; but work thus far points to a prominent role for cell surface and potentially intracellular HSPGs rather than ECM localized HSPGs.

A number of questions in this area of research remain outstanding. A definitive role of *N-* sulfation has not been established, despite some evidence for specific interactions between tau and *N-* sulfated HS glycans. In addition, there is no study on the glycan determinants of HSPG mediated tau secretion, and the mechanism by which HSPGs drive non-vesicular tau secretion is still poorly understood.

The extent to which HSPGs play a role in tau protein aggregation *in vivo* remains unclear, despite ample *in vitro* evidence of heparin induced tau aggregation. Nevertheless, the presence of HS within brain derived tau fibrils indicates that interaction between HS and tau must occur at some point during fibril assembly *in vivo*. One possibility is that cell surface HSPGs act as nucleation sites for tau aggregation under pathological conditions and are then internalized. Though evidence exists for HSPG independent tau uptake pathways under certain conditions, the receptors involved have not yet been identified. Further study of non-AD tauopathies such as PSP and by cells where HSPG independent tau uptake is observed may help elucidate the components of these pathways. Finally, looking forward toward translation, the prominent role of HSPGs in tau pathology, and the capacity of glycan-based HS analogs to inhibit tau propagation in cells suggests a potential application of HS based drugs in the treatment of AD and other tauopathies (Alavi Naini and Soussi-Yanicostas, 2018). Glycan-based drug discovery efforts will clearly benefit from a more detailed understanding of the mechanistic roles of HS-sulfation patterns in the pathogenetic mechanisms of tauopathies.

## Author Contributions

DM wrote the initial manuscript. CW and DM conceived of the review. XL and JZ contributed to the content of the figure. XL, JZ, RL, FZ, JL, and LW provided feedback and edited the manuscript. All authors contributed to the article and approved the submitted version.

## Conflict of Interest

The authors declare that the research was conducted in the absence of any commercial or financial relationships that could be construed as a potential conflict of interest.
